# Harnessing Artificial Intelligence in Obesity Research and Management: A Comprehensive Review

**DOI:** 10.3390/diagnostics15030396

**Published:** 2025-02-06

**Authors:** Sarfuddin Azmi, Faisal Kunnathodi, Haifa F. Alotaibi, Waleed Alhazzani, Mohammad Mustafa, Ishtiaque Ahmad, Riyasdeen Anvarbatcha, Miltiades D. Lytras, Amr A. Arafat

**Affiliations:** 1 Scientific Research Center, Al Hussain bin Ali Street, Ministry of Defense Health Services, Riyadh 12485, Saudi Arabia; sarfu_azmi@yahoo.co.in (S.A.); faisalbdu@gmail.com (F.K.); haifaotaibe@gmail.com (H.F.A.); waleed.al-hazzani@medportal.ca (W.A.); khan.mdmustafa@gmail.com (M.M.); isthtiaquehamdard@gmail.com (I.A.); anfariyas@gmail.com (R.A.); 2 Department of Family Medicine, Prince Sultan Military Medical City, Riyadh 11159, Saudi Arabia; 3 Critical Care and Internal Medicine Department, College of Medicine, Imam Abdulrahman Bin Faisal University, Dammam 31441, Saudi Arabia; 4 Computer Science Department, College of Engineering, Effat University, Jeddah 21478, Saudi Arabia; miltiadis.lytras@gmail.com; 5 Department of Management, School of Business and Economics, The American College of Greece, 15342 Athens, Greece; 6 Departments of Adult Cardiac Surgery, Prince Sultan Cardiac Center, Riyadh 31982, Saudi Arabia

**Keywords:** artificial intelligence, machine learning, obesity, deep learning, risk prediction, precision medicine

## Abstract

**Purpose:** This review aims to explore the clinical and research applications of artificial intelligence (AI), particularly machine learning (ML) and deep learning (DL), in understanding, predicting, and managing obesity. It assesses the use of AI tools to identify obesity-related risk factors, predict outcomes, personalize treatments, and improve healthcare interventions for obesity. **Methods:** A comprehensive literature search was conducted using PubMed and Google Scholar, with keywords including “artificial intelligence”, “machine learning”, “deep learning”, “obesity”, “obesity management”, and related terms. Studies focusing on AI’s role in obesity research, management, and therapeutic interventions were reviewed, including observational studies, systematic reviews, and clinical applications. **Results:** This review identifies numerous AI-driven models, such as ML and DL, used in obesity prediction, patient stratification, and personalized management strategies. Applications of AI in obesity research include risk prediction, early detection, and individualization of treatment plans. AI has facilitated the development of predictive models utilizing various data sources, such as genetic, epigenetic, and clinical data. However, AI models vary in effectiveness, influenced by dataset type, research goals, and model interpretability. Performance metrics such as accuracy, precision, recall, and F1-score were evaluated to optimize model selection. **Conclusions:** AI offers promising advancements in obesity management, enabling more personalized and efficient care. While technology presents considerable potential, challenges such as data quality, ethical considerations, and technical requirements remain. Addressing these will be essential to fully harness AI’s potential in obesity research and treatment, supporting a shift toward precision healthcare.

## 1. Introduction

Obesity poses a significant global health challenge due to its association with various morbidities [1,2]. Despite being commonly measured by body mass index (BMI), obesity is a complex condition influenced by genetic, behavioral, and environmental factors. Obesity is a leading cause of preventable mortality, necessitating significant social resources in both high and low- to middle-income countries [3,4]. In 2014, the World Health Organization (WHO) reported that approximately 1.9 billion individuals were overweight and over 600 million were obese. By 2030, projections indicate that 2.16 billion individuals will be overweight and nearly 1.2 billion will be obese. Therefore, a substantial portion of the global population faces increased risks of various health complications associated with excess weight [5,6].

Numerous research studies have highlighted the multifaceted nature of obesity, indicating that it is not simply a matter of excess weight but rather a complex health issue influenced by a variety of factors. These factors include individual factors such as genetics, learned behaviors, and broader societal and cultural influences such as unhealthy eating habits. While genetic and epigenetic factors contribute to obesity, lifestyle choices are a major contributor [7,8]. Factors such as low physical activity and poor dietary habits are known to heavily influence the development and progression of obesity [9].

Moreover, obesity is intricately linked to the exacerbation of pre-existing health conditions and the onset of new health-related issues. Various forms of obesity, including abdominal obesity, have been associated with an increased risk of chronic diseases such as asthma, cancer, diabetes mellitus, hypercholesterolemia, and cardiovascular diseases. Furthermore, obesity impacts several organ systems, including the cardiovascular, endocrine, central nervous, and gastrointestinal systems [10,11,12].

Artificial intelligence (AI) is revolutionizing medicine in many aspects, including diagnosis, treatment, and operational efficiency [13]. AI methods, such as machine learning (ML) and deep learning (DL), are commonly applied across the healthcare sector for research and treatment [14]. These techniques are pivotal in disease prediction, detection, and patient risk stratification. In obesity, AI models leverage diverse data modalities, which include encompassing anthropometric measurements, environmental parameters, geographical contexts, educational backgrounds, clinical profiles, genetic biomarkers, and epigenetic signatures, and untangle the complexity of big data to assist healthcare providers in formulating personalized management strategies and tailored treatment plans [14]. This strategy is already used in cancer, where AI models can recommend the best therapy combinations based on the patient’s tumor characteristics [15]. The use of AI is revolutionizing obesity treatment by enabling personalized healthcare strategies. Moreover, AI facilitates continuous monitoring and adaptive feedback, leverages wearable technology for real-time recommendations, and addresses psychological aspects through customized behavioral interventions [16].

Despite the potential benefits, ethical considerations, such as data privacy, must be carefully managed to ensure patient safety and confidentiality. While challenges exist, the integration of AI holds immense potential in reshaping obesity management paradigms. Through its capacity to process diverse data sources and provide personalized solutions, AI stands to revolutionize healthcare delivery in the fight against obesity. By addressing the systemic nature of the condition and offering tailored interventions, AI provides hope for improving patient outcomes and mitigating the global burden of obesity-related diseases (Figure—graphical abstract).

## 2. Methodology

A comprehensive review was undertaken of recent literature using PubMed and Google Scholar, with keywords such as “artificial intelligence”, “machine learning”, “deep learning”, “obesity”, “obesity management”, and related phrases. The search focused on observational studies, systematic reviews, and clinical applications to investigate AI’s potential involvement in obesity research, management, and therapeutic interventions. AI integration with anti-obesity medication, bariatric surgery, and behavioral or lifestyle changes were among the main areas of focus. This study intended to address the following questions: how might AI, specifically ML and DL, be used to understand better, predict, and manage obesity? What are the existing AI-driven models for obesity prediction and management, and how effective are they? What are the future directions, recommendations, and the problems and limitations of applying artificial intelligence to obesity research and management? How can AI be applied to clinical practice to improve obesity management? The findings regarding the effectiveness of various machine learning and deep learning models in predicting and managing obesity have typically been organized into tables for clarity.

## 3. Classification of Obesity

Obesity is often categorized based on BMI; a measure of body fat derived by dividing a person’s weight into kilograms by their height in square meters. The WHO and the National Institutes of Health (NIH) classify BMI into three classes. Class I obesity (BMI: 30.0–34.9 kg/m^2^) is defined by a moderate amount of excess body fat, increasing the risk of obesity-related disorders such as type II diabetes mellitus, hypertension, and cardiovascular disease [17]. Class II obesity (BMI: 35.0–39.9 kg/m^2^) is characterized by more excess body fat than class I obesity, which puts them at a greater risk for obesity-related health problems [18], and class III obesity (BMI ≥ 40.0 kg/m^2^) substantially increases the risk of serious health issues such as type II diabetes mellitus, heart disease, stroke, sleep apnea, and cancer [18]. Overweight or obesity in children and adolescents is classified according to BMI percentile, with overweight BMI ranging from 85 to 95 percentile, obese BMI larger than 95 percentile, and severely obese BMI greater than or equal to 120% of the 95 percentile. Childhood obesity can result in a range of short- and long-term health concerns, including type II diabetes, CVD, sleep apnea, joint problems, and psychosocial issues [19].

Although BMI is a widely used metric to categorize obesity, it fails to accurately reflect changes in the distribution of fat or body composition. Therefore, a complete picture of obesity can be obtained by incorporating additional parameters and tools such as the waist–hip ratio, body fat percentage, skinfold thickness measurement, hydrostatic weighing, air displacement plethysmography, body volume index, bioelectrical impedance analysis, and medical history. These factors led to the following categories of obesity: central or visceral obesity; an “apple-shaped” body; fat mainly around the abdomen [20]; gynoid (peripheral or subcutaneous) obesity; “pear-shaped” figure; extra fat primarily in the hip and thigh areas *;* visceral fat obesity; the buildup of fat around essential organs, including the pancreas, liver, and intestines inside the abdominal cavity (excessive visceral fat is linked to inflammation, insulin resistance, and a higher risk of heart disease, type II diabetes mellitus, and cancer) [21]; subcutaneous fat obesity; the buildup of fat just below the skin’s surface, usually in the abdomen, hips, thighs, and buttocks; and mixed (central and peripheral) obesity. A combination of peripheral (gynoid) and central (android) fat distribution patterns characterizes mixed obesity. People who have mixed obesity may have fat accumulation in both the abdomen and hip/thigh areas, displaying traits of both android and gynoid obesity. Compared to isolated forms of obesity, this type may carry a greater risk of metabolic complications [20,22].

## 4. Obesity Paradox

The term “obesity paradox” refers to the surprising discovery that, in certain groups, people who are obese may have better results than those who are normal weight or underweight, especially in the setting of certain chronic diseases or emerging health issues, such as heart failure and surgery [23]. This effect was first detected in cardiovascular disease studies [23], where researchers discovered that obese people died at a lower rate than those who were normal weight or underweight. Similar findings were observed in a variety of chronic conditions, including chronic renal disease, heart failure, and some forms of cancer [24,25,26]. These findings may challenge conventional wisdom about the hazards of obesity; they should not overshadow the well-established links between obesity and a variety of chronic disorders, nor should they discourage efforts to prevent and cure obesity.

## 5. Artificial Intelligence

Artificial intelligence describes developing computer systems that can carry out tasks that require human intelligence. After initial programming, nonhuman technologies with AI can learn and operate independently. Based on learned data inputs, these devices execute adaptive tasks and function with increasing degrees of autonomy from direct human direction. ML is an essential component of AI that improves machine performance by exposing it to cumulative data inputs. ML algorithms predict patient outcomes, screen radiology images, make personalized treatment strategies, and detect illnesses [27].

Machine learning and deep learning algorithms were first developed in the 1950s, initially sparking enthusiasm but remaining inactive for many decades [28]. The development of AI can be divided into two stages: symbolic AI (i.e., good old-fashioned AI) and modern AI (i.e., machine learning AI). The 1950s to 1980s were known as the era of symbolic AI, which attempted to imitate human-level intelligence by manually building massive sets of explicit rules to deal with knowledge. Symbolic AI solves clearly defined, logical problems such as rule-based inquiries and response systems. For example, A symbolic AI-based medical diagnostic system leverages a knowledge base of explicitly defined rules to infer and deduce plausible diagnoses from the symptoms reported by a patient. For instance, the system may employ a rule that states: IF the patient exhibits a fever, a cough, and difficulty breathing, THEN the patient may be suffering from pneumonia. This rule-based reasoning allows the system to logically analyze the presented evidence and determine potential medical conditions affecting the patient. The advantages of this system are the interpretability of the results and the logic behind the results can be traced. Still, it struggles with more complex, ambiguous jobs such as image categorization, speech recognition, and translation [29]. With the re-emergence of advanced computational power, ML and DL have experienced a resurgence in popularity compared to symbolic AI [28].

Machine learning has several advantages over traditional statistical methods. ML is a model-free philosophy that does not require prior statistical assumptions. The model can deal with non-linear and colinear data and handle sparse and high-dimensional data with high prediction ability. Furthermore, ML trains on part of the data (training dataset) and tests its performance on another part (testing dataset). This process makes out-of-sample prediction for ML algorithms high compared to traditional statistical methods, which are prone to overfitting. Dealing with non-structure data (medical images, words, multimedia) is another advantage of ML [30,31].

**Figure 1:**
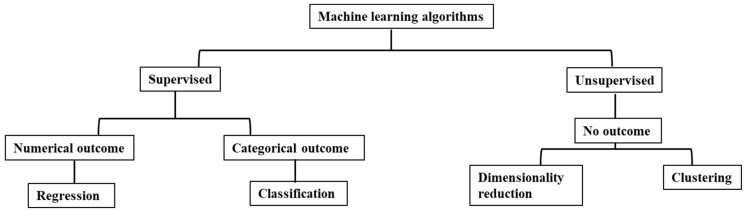
Machine learning algorithm and subcategories.

ML algorithms have three main subcategories (supervised, unsupervised, and reinforcement learning) (Figure 1). Unsupervised ML involves analyzing and clustering unlabeled datasets to uncover hidden patterns or groupings. Its ability to identify similarities and differences in data makes it particularly useful for exploratory data analysis. Unsupervised ML models are employed for clustering and dimensionality reduction tasks. On the other hand, supervised ML uses a training set containing input–output pairs to teach the algorithm to learn a function that maps inputs to outputs. The key distinction between supervised and unsupervised ML is that the former requires labeled data (input–output pairs), while the latter only requires inputs (unlabeled data). Supervised ML models are utilized for tasks such as classification, which involves assigning data to specific categories (e.g., diabetic or nondiabetic), and regression, which entails learning the relationship between input features and continuous outcomes, such as BMI. Reinforcement learning is currently integrated into robotics engineering, and its application in medical research has yet to be well-developed [30].

**Figure 2:**
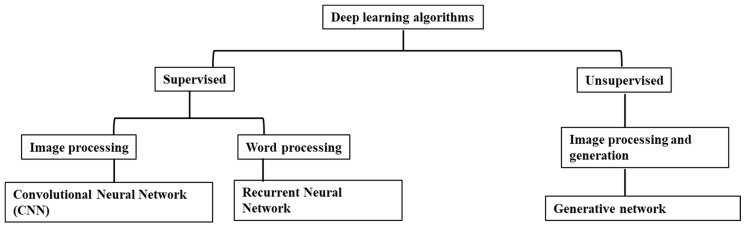
Deep learning algorithm and subcategories.

Deep learning has the potential to deal with images (convolutional neural networks (CNNs), process words (recurrent neural networks (RNNs)), and generate images (generative adversarial networks) (Figure 2).

## 6. Machine Learning Steps

**Figure 3:**
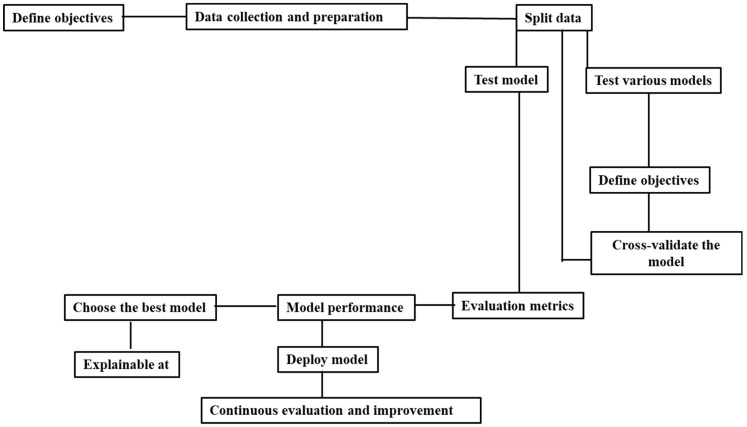
Steps involved in machine learning model generation, testing, and deployment.

Almost all supervised ML or DL models involve similar steps for practical use in healthcare research and treatment to improve patient outcomes and decision-making processes. These steps are **defining objectives** (clearly define the research question or clinical problem you want to address, such as disease diagnosis, prognosis prediction, or treatment recommendation), **data collection** (gather relevant healthcare data from sources such as electronic health records (EHRs), medical imaging, genetic databases, or clinical trials), **data preprocessing** (clean the data, handle missing values, normalize features, and encode categorical variables to prepare for modeling), **split data** (divide the dataset into training and testing sets to train and evaluate the model’s performance), **training the model** (use the training data to train the respected model, adjusting hyperparameters, cross-validating the model), **validating the model** (assess the model’s performance on the testing data using metrics such as accuracy “the proportion of correct predictions out of the total number of predictions”, precision “True positive (TP)/(TP + false positive(FP))”, recall “TP/(TP + false negative (FN))”, area under the receiver operator curve (aucROC) “model’s ability to distinguish between positive and negative classes” and F1-score “2* (Precision ∗ Recall)/(Precision + Recall”), **interpreting results** (analyze feature importance to understand which variables influence predictions and gain insights into disease mechanisms or treatment responses using explainable AI methods), **deploying the model** (if the model performs well, deploy it in clinical settings to assist healthcare professionals in decision-making), and **monitoring and updating** (continuously monitor the model’s performance and update it as needed with new data or changes in healthcare practices) (Figure 3) [30].

## 7. Artificial Intelligence in Obesity and Associated Risk Prediction

As the overweight and obesity rates increase, there is a growing need for computational tools to predict obesity to aid individuals in managing their daily meal planning routines effectively. To address obesity in its early stages, researchers and healthcare professionals are utilizing abundant datasets on obesity collected from various channels, such as electronic medical records, insurance databases, and mobile apps. Analyzing these data allows for extracting valuable insights aimed at preventing and managing obesity at an early phase [32,33]. Healthcare and researchers utilize various approaches to develop diagnostic and prognostic predictive models for biomedical applications, harnessing the high potential of ML or DL [34,35].

Numerous studies have employed ML or DL techniques to predict obesity using diverse datasets. We endeavored to comprehensively discuss the most significant and pivotal studies where ML or DL was applied in predicting obesity across various parameters, identifying the most appropriate models for different types of obesity research. In 2015, Dugan and colleagues assessed various ML models to determine the most effective model for predicting the onset of obesity in early childhood [34]. In this study, six models, namely, random tree, random forest, Iterative Dichotomiser 3 (ID3), J48, naive Bayes, and Bayes net, were utilized to construct a clinical dataset of a pediatric clinical decision support system named CHICA (Child Health Improvement via Computer Automation) [34,36]. The authors examined 167 attributes collected before a child’s second birthday for each patient. The authors evaluated the models’ accuracy using sensitivity, specificity, positive predictive value (PPV), negative predictive value (NPV), and overall accuracy. Among the six models, the random tree and ID3 models were the most sensitive for predicting obesity. The most influential feature observed was being overweight before age two, especially if the child was not overweight before their first birthday but became overweight between one and two years old. Additionally, protective factors such as being very tall before six months and influential factors such as not being overweight before two years but using a walker and being white were also identified. This study provides valuable insights for clinicians in delivering targeted interventions during critical developmental stages, potentially preventing the onset of obesity.

In another study, Allen and collaborators investigated the interaction effect of ecosystems on the development of obesity in adolescents [37]. In this study, the authors explored the interaction features of a complex multilevel environment that reinforces obesity/obesogenic behaviors through random forest ML models to predict obesity in young people. In this study, the authors used data from the ABCD study (https://abcdstudy.org), which was collected from 22 different sites in the USA. The primary outcomes of this study were age- and sex-adjusted waist-to-height ratio z scores. The investigators used 120 features as predictors of each child’s waist-to-height ratio. The study used an explainable AI approach, which helps to discover the interaction between obesogenic features of the multiple environments that youth navigate. The analysis revealed that children from less educated households had higher waist-to-height ratio z scores when living in neighborhoods with fewer adults holding high school diplomas. Similarly, those from low-income homes showed elevated z scores in areas with higher poverty rates. Additionally, children in low-income households had higher z-scores if their neighborhoods had lower particle pollution levels. Children residing in low-income neighborhoods exhibited higher waist-to-height ratio z-scores if they engaged in less than 23 min of sports per week. Furthermore, children in communities with a low proportion of adults holding high school diplomas had higher z-scores if they lived in areas with high median home values. In contrast, those in neighborhoods with a high percentage of households below the poverty line had elevated z-scores if they resided in areas with numerous single-parent households.

Kaur and coworkers investigated the application of ML to predict obesity risk and plan meals to reduce obesity. They utilized various ML algorithms, including gradient boosting (GB), bagging meta-estimator (BME), XGBoost (XGB), random forest, SVM, and K-nearest neighbor (KNN) [38]. Their dataset, sourced from the UCI Machine Learning repository, contained features such as physical descriptions, meal calorific values, and eating habits. Across different training and testing ratios (90:10, 80:20, 70:30, and 60:40), they found that GB achieved the highest accuracy (98.11%) at a 90:10 ratio. At an 80:20 ratio, GB and XGB showed similar accuracy levels (97.87% and 97.79%, respectively). Additionally, the SVM and KNN algorithms performed poorly compared to the other algorithms, with a consistent trend of accuracy observed across all ratios.

Furthermore, several studies have used genetic variation in the prediction of obesity or obesity risk using the ML or DL method. In 2018, Wang and collaborators utilized genetic variations identified through next-generation sequencing (NGS) to predict obesity risk by employing ML models such as SVMs, KNNs, and decision trees. Their study incorporated 139 single nucleotide polymorphisms (SNPs) alongside age and sex data. They employed multivariate logistic regression to assess the importance of these selected features. By employing stepwise multivariate logistic regression, they identified nine SNPs for designing obesity prediction models using ML techniques. The SVM model emerged as the most effective classifier, achieving notable performance metrics: 70.77% accuracy, 80.09% sensitivity, and 63.02% specificity. This investigation underscores the efficacy of the selected SNPs in detecting obesity risk, highlighting the potential of ML-based methods for preliminary analyses of genetic predispositions to obesity [39]. We summarize the literature on ML and DL models to predict obesity or related factors in Table 1.

## 8. Artificial Intelligence in the Management of Obesity

Treatment and management of obesity entail all-encompassing approaches to enhance general health, minimize extra body weight, and avert obesity-related issues. There are several essential points, such as lifestyle modifications (balanced diet, physical activity, sleep, and wake), behavioral therapy (managing stress, emotional triggers for overeating, sedentary life, etc.), pharmacotherapy (suppressing appetite, reducing nutrient absorption, altering metabolism, etc.), and bariatric surgery (Figure—graphical abstract). Healthcare practitioners can assist people in reaching and maintaining a healthy weight while lowering their risk of obesity-related problems by combining lifestyle changes, behavioral therapy, medication, and, if necessary, surgical procedures [65,66].

Artificial intelligence has the potential to transform obesity treatment and management by implementing innovative strategies to address this intricate health challenge, as outlined in Table 2. AI algorithms are being used to create personalized interventions, prediction models, and decision support systems for healthcare personnel [49,67]. These technologies use large databases to uncover trends, predict obesity risk factors, and offer personalized treatment regimens based on individual features and health history. Additionally, AI-powered solutions enable remote monitoring of patients, allowing for rapid interventions and alterations to treatment regimens. By leveraging AI, healthcare professionals can increase the efficiency and effectiveness of obesity management, resulting in better patient outcomes and lower healthcare expenditures [16].

Moreover, AI technologies can support the ongoing management of obesity by facilitating continuous monitoring and feedback. Wearable devices that track physical activity, sleep patterns, and caloric intake can feed data into AI systems for real-time analysis. Recommendations can then be adjusted dynamically, providing individuals with immediate guidance to help them make healthier choices and maintain progress toward their weight management goals. Intelligent systems can also offer behavioral interventions, addressing one of the critical challenges in obesity management [68]. For instance, interventions might include AI-powered applications that incentivize healthier food choices or more active lifestyles. The AI app generates dietary recommendations that suit the user’s dietary preferences and type. These suggestions are customized based on the user’s input, providing diverse meal options matching their diet. The user’s information is processed to refine the model for better recommendations. Additionally, AI can help individuals identify psychological patterns that contribute to obesity and suggest behavioral modification techniques to help individuals cope with cravings and triggers for unhealthy eating behaviors. Likewise, the app collects user information on physical fitness by evaluating their fitness goals, BMI, age, and overall health condition [70]. Through interaction, the app gains insights into users’ exercise preferences, past fitness endeavors, and any specific health issues to deliver customized recommendations. This personalized strategy seeks to provide suggestions that closely match each user’s requirements and preferences, thereby improving the app’s overall effectiveness and user satisfaction.

However, while AI holds significant promise in reshaping obesity treatment and management, it is crucial to ensure that these technologies are developed and implemented with careful consideration of ethical concerns such as data privacy, the potential for biased algorithms, and the accuracy of AI-based recommendations [71]. Maintaining a transparent, responsible approach to integrating AI in healthcare is essential for realizing its full potential while safeguarding patient well-being and trust.

## 9. Anti-Obesity Pharmacotherapy and Artificial Intelligence

Obesity involves complex metabolic and neurohormonal processes. Hunger and satiety regulation encompass mechanisms within the central nervous system (CNS), peripheral nervous system, and hormonal pathways. Hormones and signals from vagal afferent neurons modulate hunger and satiety by responding to mechanical changes, the presence of macronutrients, and alterations in pH and tonicity. For instance, ghrelin originates from stomach fundic cells and stimulates hunger by activating AgRP/NYP neurons. At the same time, glucagon-like peptide-1 (GLP-1), released by the gut after food intake, induces satiety by activating POMC/CART neurons and inhibiting AgRP/NYP neurons. Insulin and leptin similarly contribute to appetite suppression. There are five FDA-approved anti-obesity medications: orlistat (which acts on the gastrointestinal tract and inhibits the absorption of fatty acids) [72], phentermine (which functions as an adrenergic agent, decreasing hunger) [73], phentermine–topiramate (which reduces the desire to eat by boosting dopamine), naltrexone–bupropion (which acts as naltrexone; which suppresses appetite, and bupropion is a dopamine and norepinephrine reuptake inhibitor) [74], liraglutide (which is an agonist of GLP-1), and semaglutide (which activates the GLP-1 receptor) [75].

Obesity treatment involves a variety of techniques, including lifestyle changes, surgery, and medications. Lifestyle modifications sometimes require significant commitment, and surgery is expensive and complex; therefore, medicine is the preferred option for many people due to its accessibility and less invasive nature. AI is rapidly evolving and could revolutionize how we diagnose and treat obesity. In the realm of drug reactions, the typical ML approach involves several steps, such as cohort selection, data processing, predictor identification, creating and validating machine learning models, assigning subgroups, and analyzing drug responses. Despite its potential, current research needs to provide more proof to integrate ML algorithms into everyday clinical practice due to complexity, validation issues, and unclear efficacy. However, as evidence accumulates, we anticipate ML will increasingly influence precision pharmacotherapy for treating obesity.

## 10. Artificial Intelligence and Bariatric Surgery

Bariatric surgery encompasses a range of surgical interventions aimed at aiding individuals with severe obesity in weight loss. These procedures function by limiting stomach capacity, modifying digestion, or combining both approaches. Typical bariatric surgeries include gastric bypass, sleeve gastrectomy, adjustable gastric banding, and biliopancreatic diversion with duodenal switching [76]. Conversely, non-bariatric surgery encompasses any surgical intervention unrelated to weight reduction. These procedures vary from corrective surgeries addressing specific medical issues like cardiac or orthopedic conditions to elective treatments such as cosmetic enhancements. Fundamentally, non-bariatric surgeries do not target obesity treatment as their primary objective.

Artificial intelligence algorithms have been integrated into every stage of the perioperative process for patients undergoing bariatric surgery (BS), from presurgical assessment and risk evaluation to predicting postoperative complications and outcomes [77]. Thorough preoperative evaluation is vital for bariatric surgery candidates to gauge their risks and outlook. The objective is to pinpoint obesity-related health issues, identify high-risk patients, and reduce postoperative complication risks. Numerous studies indicate that AI has proven to be a valuable tool in this process. For example, Zohou and collaborators investigated six ML models to anticipate challenging intubation in obese patients and identified three effective approaches. Among these algorithms, the extreme gradient boosting algorithm achieves an accuracy exceeding 80% and a precision reaching 100% [69].

Obstructive sleep apnea (OSA) is a serious sleep disorder in which breathing repeatedly stops and starts during sleep due to throat muscle relaxation. Polysomnography (PSG) is widely recognized as a reliable and efficient diagnostic method, offering insights into the severity of OSA and the extent of sleep disturbances [78]. Treatment options include lifestyle changes such as weight loss, exercise, and continuous positive airway pressure (CPAP) machines to maintain open airways during sleep [79]. Oral appliances such as mandibular advancement devices are also used [78,79]. Surgery is considered in selected patients but is not typically the first choice due to potential risks and limited evidence of effectiveness. Furthermore, patients with obesity commonly experience lung dysfunction, including conditions such as chronic obstructive pulmonary disease (COPD), chronic lung disease, and asthma, which can be detected through spirometry. Assessing these conditions during preoperative evaluation is crucial. Viswanath and associates evaluated smartphone spirometry efforts and found that neural networks can extract more information from signals than traditional methods, enabling expert-level validity feedback for smartphone-based spirometry [80]. However, we could not find randomized trials comparing AI to traditional perioperative evaluation methods.

Artificial intelligence has a variety of possible applications during the intraoperative phase, including medication management, hemodynamic optimization, neuromuscular block monitoring, and anesthesia depth monitoring. However, its application in bariatric surgery is largely unknown. To the best of our knowledge, there is little literature on the use of ML in this stage, such as Ingrande et al.’s study predicting the early distribution kinetics of propofol [81], Twinanda and colleagues’ estimation of surgery duration and quality improvement [82], and automatic identification of steps in laparoscopic sleeve gastrectomy from operative video with a high degree of accuracy [83].

Complications after bariatric surgery may emerge in various forms and at different times, ranging from surgical complications such as bleeding and bowel obstruction to issues within the pulmonary system, such as pneumonia and thrombosis, as well as nutritional, hepatobiliary, gastrointestinal, and neurological problems. Some research has explored the use of ML in predicting potential postoperative complications following bariatric surgery. Taheri and associates designed artificial neural networks that predict postoperative complications at ten days, one month, and three months by considering patient age, BMI, smoking status, intraoperative complications, associated health conditions, laboratory results, and ultrasound and endoscopic examination findings. Their results demonstrated the predictive system’s substantial accuracy, specificity, and sensitivity in identifying complications [84]. Additionally, Nudel and coworkers employed two ML models, ANN and XGB, to predict complications such as leakage and deep vein thrombosis following bariatric surgery. Their analysis indicated that XGB and the ANN outperformed the LR in predicting leakage and venous thromboembolism [85].

Additional studies have explored ML and DL applications to predict complications related to bariatric surgery’s perioperative, intraoperative, and postoperative periods. We have compiled a summary table to provide an overview of this literature (Table 3).

## 11. Recent Advances in AI in Obesity

Artificial intelligence applications in diagnosing and managing obesity are rapidly evolving. Several technological advancements have been integrated into the obesity management armamentarium, including deep learning, digital twins, and the application of explainable artificial intelligence methods. Deep learning methods have several applications in obesity detection and management. Deep learning, particularly convolutional neural networks, can be used for medical image analysis to assess fat distribution and obesity-related disease [91,92]. Furthermore, deep learning can be used to predict and untangle complex relationships and interactions between genetic data, demographics, and lifestyle changes; consequently, it can enhance early detection and interventions [45,46]. Deep learning can be integrated into wearable technology to track activities, sleep patterns, and heart rate. Therefore, personalized recommendations on obesity management can be provided [93,94]. Other applications of deep learning include behavioral analysis, nutritional assessment, and enhancing telemedicine and personalized interventions [95,96].

Digital twin technology has a promising application in obesity management by creating digital replicas of individuals’ physiological and behavioral profiles, allowing for personalized monitoring and management strategies for obesity. Furthermore, digital twins can simulate the effects of various interventions on an individual’s weight and health metrics, enabling tailored recommendations [97].

Machine learning methods are generally a black box and the explanation of how the model predicted the outcome is not always clear. Therefore, explainable AI methods have emerged to provide explanations for machine learning models and which features contributed mainly to the prediction. Several explainable AI methods can be applied to explain the model output, such as LIME (Local Interpretable Model-agnostic Explanations), feature importance, SHAP (SHapley Additive exPlanations), and Quantum Lattice. These methods provide details on which variables contributed mostly to model output, the direction of effect, and the interaction between different features [98,99].

## 12. Current Limitations, Future Directions, and Recommendations

The future of AI in obesity research and management is promising, and poised to revolutionize personalized therapies, diagnostics, and treatment approaches. Wearables enable AI to build individualized nutrition programs, forecast obesity onset, and provide continuous monitoring. It will improve behavioral therapy, medication research, and public health campaigns by analyzing large databases to identify more successful techniques. AI’s incorporation into surgical procedures and smart gadgets will improve outcomes, while its use in environmental treatments and VR/AR apps will encourage healthy lifestyles. These developments will result in more precise, effective, and scalable solutions for obesity, ultimately enhancing public health and individual well-being [29].

While AI and machine learning (ML) have the potential to transform obesity management, several challenges limit their broad implementation. A major concern is data quality, as AI models require large, high-quality datasets that incorporate diverse demographic, genetic, and behavioral information. Many current models suffer from incomplete or inconsistent data, which decrease their accuracy and limit their applicability across different populations [14,100]. Additionally, the technical infrastructure needed to support ML, including powerful computing systems and extensive data storage, poses a significant barrier, particularly in resource-constrained healthcare settings.

Organizational capacity and expertise present another limitation. Successfully integrating AI into clinical workflows requires that healthcare organizations have staff skilled in AI and ML, which remains a hurdle for many institutions. Ethical considerations also demand attention; AI systems in healthcare must be designed with transparency, fairness, and robust privacy protections [14,101]. Given the sensitive nature of health data, strict measures are needed to prevent misuse or unauthorized access, adding further regulatory and ethical challenges.

Addressing these limitations will require the development of robust data collection protocols to improve the consistency and completeness of datasets. Standardizing data collection processes will enhance the reliability of AI models in managing obesity. Building partnerships across institutions to create shared data repositories can increase sample diversity and size, improving model training and applicability. Investments in technical infrastructure and collaborations with technology firms can help support healthcare organizations in adopting AI/ML solutions, extending the benefits of these advancements to more facilities. To address organizational challenges, training programs for healthcare providers can build proficiency in AI/ML applications. Educational initiatives should emphasize understanding AI-generated insights and effectively integrating them into patient care. Additionally, expanded ethical guidelines and regulatory frameworks specific to AI in healthcare are essential. Such guidelines should focus on patient consent, transparency, and model interpretability to foster accountability and build trust.

To enhance obesity prevention and management, healthcare systems should integrate a variety of datasets, including electronic medical records and mobile health applications, to improve predictive models and facilitate early interventions. It is vital to implement targeted strategies, such as researching at-risk populations, utilizing machine learning for personalized meal planning, and incorporating genetic data into risk assessments. Developing AI-driven tools for ongoing monitoring and prioritizing ethical AI practices will help sustain patient trust. Collaboration among healthcare professionals, data scientists, and policymakers is crucial for creating comprehensive management strategies that encompass lifestyle changes and advancements in bariatric surgery. Furthermore, training healthcare providers in using AI tools and supporting research on the effectiveness of these models will enhance obesity management efforts and improve patient outcomes. Key observations and recommendations from the recent literature review are summarized in Table 4.

## 13. Conclusions

This review highlights the transformative role of artificial intelligence in obesity research and management, encompassing predictive modeling, personalized intervention strategies, pharmacotherapy, and surgical support. With the integration of machine learning and deep learning algorithms, AI has demonstrated significant potential in predicting obesity risk, managing treatment pathways, and providing real-time, tailored recommendations. These innovations are particularly promising in advancing personalized healthcare and addressing the multifaceted challenges of obesity, from genetic predispositions to environmental and behavioral influences.

However, while AI offers powerful tools to combat obesity, realizing its full potential will require overcoming key challenges, such as ensuring data quality, safeguarding patient privacy, and maintaining ethical standards. Further research and refinement are essential to develop robust, scalable AI solutions that not only enhance patient outcomes but also support healthcare practitioners in delivering equitable and efficient care. Ultimately, AI-driven insights and interventions provide a promising path forward in addressing the global obesity epidemic, offering hope for improved health outcomes and a substantial reduction in obesity-related health burdens.

## Figures and Tables

**Table 1:** Summary of studies that used machine learning and deep learning models to predict obesity and related risk factors.

Authors	Objective	ML or DL Models Tested and Dataset Used	Outperformed Model
Almeida et al. [40]	It aimed to develop and validate predictive models for percentage body fat based on anthropometric data other than skinfold thickness.	NN and MLM compared with various previous studies. Data from 3084 pre-pubertal children. NN excels in classifying ‘normal’ and ‘obese’ categories; however, significant differences appeared in the following: under fat—NN (18.8%) vs. MLM (43.1%); overfat—NN (56.7%) vs. MLM (58.7%).	
Carvalho et al. [41]	ML in Mission Kids can create a knowledge base by analyzing workout data to recognize effective performance, aiding in combating childhood obesity through personalized feedback and tailored exercise recommendations.	DT (J48), RF, and MLP. Data: 30 children who performed various physical exercises. Average classification accuracy rate for various exercises. DT: 91.52%. RF: 92.95. MLP: 74.76.	RF outperformed in the evaluation of the correct exercise.
Chen et al. [42]	This study aimed to use blood measurements to detect overweight conditions and identify key factors distinguishing overweight from healthy individuals.	ELM, SVM, and BPNN. Data: Blood and biochemical data of 225 obese and 251 healthy individuals. ELM: 89.98% AUC, 83.95% Sen, and 96.02% Spec. SVM: ~89.5% AUC, ~82% Sen, and ~97% Spec. BPNN: ~83.5% AUC, ~83.5% Sen, and ~84% Spec.	ELM outperformed SVM and BPNN.
Dugan et al. [34]	This study sought to predict obesity in children after age two using data gathered from the CHICA clinical decision support system.	RT, RF, ID3, J48, naive Bayes, BayesNet. Data: CHICA dataset. RT: 84, 88, 80% (ACC, Sen, Spec). RF: 86, 86, 85. ID3: 84, 88, 82. J48: 79, 82, 76. NB: 63, 58, 69. BN: 63, 58, 68.	RT and ID3 were more sensitive in predicting obesity.
Dunstan et al. [43]	This study aimed to predict country-level obesity prevalence using national sales data for specific food and beverage categories, utilizing three non-linear regression machine learning methods.	SVM, RF, and XGB. Data: 48 categories of food and beverages across 79 countries and obese adult populations in these countries Root mean square error (RMSE). SVM: 0.063. RF: 0.057. XGB: 0.058.	RF showed the best execution and was closely followed by XGB.
Ergün et al. [44]	This study aimed to develop an automated system for detecting and monitoring obesity by classifying affected areas based on diverging arteries and BMI.	LR, MLP. Data: Divergent arteries and BMI of 30 healthy and 52 obese individuals. LR: 88.4% Sen and 86.6% Spec. MLP: 92.3% Sen and 86.6 Spec.	MLP performed better than LR.
Gerl et al. [45]	The objective of this work was to use advanced ML modeling to predict several indicators of obesity based on the plasma lipidome in a large population cohort.	RF, GB, Lasso and Cubist models Data: Plasma lipidome of 1061 participants of the FINRISK 2012 population cohort. Lasso regression: Mean absolute error (MAE) of 3.61 ± 0.33, explaining 73% of the variation in BFP.	Lasso and Cubist models showed better performance.
Heydari et al. [46]	This study aimed to classify obesity using artificial neural networks and logistic regression, considering socio-economic status and anthropometric measures.	LR and NN. Data: Socioeconomic status and anthropometric measures of 414 healthy military personnel in southern Iran. LR: 80.2% Sen, 81.9% Spec, 0.888 ROC. NN: 79.7% Sen, 83.7% Spec, 0.884 ROC.	LR and NN showed comparative overall performance.
Jindal et al. [47]	Utilized the R ensemble prediction model and Python interface to propose an ensemble ML approach for predicting obesity.	RF and partial least squares (PLS). Data: Dataset containing several parameters related to obesity. Both RF and PLS predicted obesity with an accuracy of 89.68%.	
Kaur et al. [38]	Two separate datasets were used to predict obesity and necessary meals in adulthood using various ML algorithms.	GB, XGB, BME, SVM, KNN. Data: UCI Machine Learning Repository of 2111 records with 16 attributes; % of the accuracy of prediction XGB (97.79), GB (97.16), SVM (87.7), and KNN (82.3).	GB and XGB performed very well in prediction.
Kupusinac et al. [48]	This study employed ANN to understand the relationship between fat and fat-free mass for diagnosing obesity and predicting its associated comorbidities.	ANN compared with previous formulas. Data: 2755 subjects with ages from 18 to 88 y and BMI from 16.60 to 64.60 kg/m^2^. ANN predicted with an accuracy of 80.43 ± 1.48, which increased average predictive accuracy from +1.23% to +3.12% with previous formulas.	ANN performed better in terms of accuracy.
Lin et al. [49]	Employed a user-friendly ML system to screen overweight individuals and predict obesity variables.	LR, KNN, ANN/MLP, DT, RF, GBM, CatBoost. Data: Age, sex, medical history, and habits of 5236 Chinese participants. Metrics and score for predicting overweight to obesity: LR (0.84–0.85), KNN (0.87–0.82), ANN (0.88–0.83), DT (1–0.79), RF (1–0.84), GBM (0.87–0.84), and CatBoost (0.91–0.83).	CatBoost demonstrated a comparatively higher accuracy.
Lingren et al. [50]	This study aimed to develop a model to precisely identify severe early-onset childhood obesity in children aged 1–6 years using EHR data.	SVM and NB, in combination with WEKA data mining software (V 3.6). Data: EHR databases from the Boston Children’s Hospital (BCH) and Cincinnati Children’s Hospital and Medical Center (CCHMC). NB: 0.9–0.95 PPV. SVM: 0.733–0.813 PPV.	In terms of PPV, NB performed better than SVM.
Machorro-Cano et al. [51]	This paper presented PISIoT, an advanced smart health platform that leverages ML and IoT technologies to prevent, detect, treat, and manage being overweight, obesity, and other related health disorders.	Weka API and J48 were used to identify critical variables and classify patients. Apache Mahout and RuleML were used to generate medical recommendations. Data: 40 elderly obese participants aged 60 to 80 years. Data for each patient were based on 17 predictor attributes like calories consumed and burned, activity minutes, sleep duration, weight, etc.	J48 identified critical variables like physical activity, heart rate, weight, and calorie intake in classifying obesity and associated risks.
Maharana et al. [35]	Provided a uniform method for quantifying built-environment characteristics and their relationship with obesity prevalence, allowing for cross-study comparisons.	CNN. Dataset: 150,000 high-resolution images from Google Static Maps. CNN identified 125 built environment features linked to obesity prevalence. Features like greenery, road type, housing density, etc., explained 64.8% of the variation in obesity prevalence.	This study utilized CNN to extract built-environment elements from satellite images for health indicators analysis.
Milla Kibble [52]	It aimed to generate new hypotheses about multi-molecular interactions in obesity development; large multivariate datasets were analyzed using group factor analysis (GFA).	GFA. Data: 43 monozygotic twin pairs, 25 pairs with weight discordant (δBMI > 3). GFA identified 38 components linking variables across datasets like clinical data (42), cytokine data (71), genomic data (1587), methylation data (1605), and dietary data (63).	
Montañez et al. [53]	This paper presented a genetic profile study using ML algorithms to predict the future risk of complex diseases, like obesity, based on the subjects’ SNP arrays and BMI status.	GB, KNN, SVM, RF, and multilayer perceptron neural network. Data: Database of Personal Genome Project (PGP) and manually curated database of SNP. SVM: 90.5 Sen, 88.24 Spec, 86.96 AUC. RF: 52.9 Sen, 95.6 Spec, 87.9 AUC. KNN: 64.7 Sen, 91.3 Spec, 88.6 SUC.	SVM achieved the best performance.
Nasrollah et al. [54]	This study aimed to apply ML to evaluate the utilization of childhood clinical variables and genetic risk factors in predicting adult obesity.	GB. Data: 2262 participant forms. Cardiovascular risk in YFS (Young Finns). GB: Identified SNP WGRS19 with AUC = 0.74 to 0.78 (higher WGRS19 linked with higher BMI at 9 and WGRS97 at 6 years).	
Pang et al. [55]	This study intended to predict the onset time of early childhood obesity using XGBoost through an analysis of roughly 11 million pediatric clinical interactions.	XGBoost. Data: Pediatric Big Data (PBD) repos. XGB recall for 24–36 months was 97.63%; for 72–84 months, it was 48.96%.	
Pereira et al. [56]	This study intended to apply ML algorithms to predict obesity and related disorders in the Indian population and discover significant indicators for early disease detection to enhance public health.	LR, NB, DT, KNN, RF, AdaBoost. Dataset of lifestyle, lipid profile, and symptoms tests collected through a Google form from India. RF (lifestyle test) AUC values 0.979. AdaBoost (lipid profile) AUC 1.00. RF (symptom test) AUC 1.00 for disease prediction like diabetes, hypertension, CVD, etc.	RF and AdaBoost algorithm gave better accuracy for obesity prediction.
Pouladzadeh et al. [57]	This research proposed an assistive calorie measurement system to aid patients and clinicians in their fight against diet-related health problems.	CNN. Data: 30 categories of the food image dataset (apple, banana, pasta, etc.). The recognition rate for calories (single food portions) was nearly 99–100%.	100% accuracy of food recognition.
Rajput et al. [58]	This work utilized ML and DL on publicly available i2b2 clinical datasets to identify chronic illness status, specifically obesity.	PART, NB, RF, and Hoeffding tree. NLP text mining based on the deep neuronal model and multichannel CNN. Data: i2b2 dataset. Obesity identification accuracy: PART (95.2%), NB (70.52%), RF (81.01%), Hoeffding trees (84.26%).	PART, NB, RF, and Hoeffding trees performed well with extensive feature engineering, while CNN ensembles excel due to their automatic feature learning.
Scheinker et al. [59]	Exploring variation in US county-level obesity prevalence rates using epidemiologic and ML models.	LR, GBM, RF, and NN. Data: EHR of urban emergency, US. RF: 0.85 AUC, 0.76 Sen, and 0.8 Spec. GBM: 0.83 AUC, 0.74 Sen, and 0.78 Spec. LR: 0.78 AUC, 0.7 Sen, and 0.75 Spec. NN: 0.81 AUC, 0.72 Sen, and 0.71 Spec.	RF and GB best-performing ML models.
Shao et al. [60]	This paper presented unique intelligent hybrid methods for obtaining fewer explanatory variables; the proposed forecasting models can effectively predict the body fat percentage (BFP).	MR, ANN, MARS, and SVR. Dataset: Cleveland Heart Disease dataset. DT: 78.6 Acc, 79.3 Pre, and 78.7 recall. RF: 82 Acc, 83.4 Pre, and 81.9 recall. SVM: 83.6 Acc, 84.2 Pre, and 83.6 recall. Hybrid: 85.3, 85.7, and 85.3 percent.	All hybrid models MR, ANN, MARS, and SVR performed well in BFP prediction.
Singh et al. [61]	This study aimed to predict future BMI using past BMI data from the Millennium Cohort Study, evaluating various regression and neural network models for forecasting teenager BMI.	Linear SVM, quadratic SVM, decision tree, MLPFFANN. Dataset: Millennium Cohort Study. MLPFFANN is the most effective in prediction, with an accuracy of 93.4%, with the lowest mean absolute error.	MLPFFANN had the highest accuracy.
Uçar et al. [62]	This work aimed to reliably determine BFP utilizing hybrid ML approaches with minimal parameters, based on 13 anthropometric data points.	MLFFNN, DT, SVM, and combination. MLFFNN+DT, MLFFNN+SVM, DT+SVM, MLFFNN+DT+SVM. Dataset: Anthropometric and body fat percentage values of 252 individuals.	MLFFNN and MLFFNN+DT+SVM performed well in every level of feature selection.
Zhang et al. [63]	This investigation aimed to assess the significance of non-linear information on the prediction of childhood obesity.	LR, DT, NN, Bayesian, SVM. Dataset: Wirral database. Bayesian (35.5% Sen and 91.5% Spec) and SVM (46% Sen and 72.5% Spec).	SVM showed better sensitivity, and Bayesian performed better overall.
Zheng et al. [64]	To assess four ML models for predicting obesity in Tennessee high school students using nine behaviors from the 2015 Youth Risk Behavior Surveillance System (YRBSS).	BLR, IDT, (KNN), ANN Dataset: 2015 survey data from the biennial YRBSS, Tennessee. IDT (80.23% ACC and 90.74% Spec). KNN (88.82% ACC and 93.44% Spec). ANN (84.22% ACC and 99.46% Spec).	IDT, KNN, and ANN performed much better than BLR. KNN showed the highest accuracy, and ANN, the highest specificity.

Acronyms: AdaBoost, adaptive boosting; ANN, artificial neural network; BLR, BayesNet: Bayesian network, binary logistic regression; BME, bagging meta-estimator; BPNN, backpropagation neural network; CatBoost, category boost; CNN, convolutional neural network; DT, decision tree; ELM, extreme learning machine; GB, gradient boosting; GFA, group factor analysis; ID3, Iterative Dichotomiser; IDT, improved decision tree; KNN, K-nearest neighbor; LR, logistic regression; MARS, multivariate adaptive regression spline(s); MLFFNN, multilayer feedforward neural network; MLM, multinomial logistic model; MLPFFANN, multilayer perceptron feed forward artificial neural network; MLP, multilayer perception; MR, multiple regression; NB, naive Bayes; NLP, natural language processing; NN, neural network; PART, partial C4.5 decision tree; RF, random forest; RT, random tree; RuleML, rule markup language; SVM, support vector machine; SVR, support vector regression; WEKA, Waikato Environment For Knowledge Analysis; XGB, extreme gradient boosting.

**Table 2:** AI-/ML-driven services in obesity management.

Service/Tool	Description	Purpose	AI/ML Model
CHICA pediatric obesity prediction [34]	Predicts childhood obesity based on early life data (e.g., weight trends)	Early obesity prediction	Random tree, ID3
ABCD adolescent obesity prediction [37]	Predicts obesity risk in adolescents by analyzing socioeconomic and environmental factors	Socio-environmental risk prediction	Random forest, explainable AI
Meal planning and obesity risk reduction [38]	Uses ML to suggest personalized meal plans based on caloric needs, lifestyle habits	Personalized meal recommendations	Gradient boosting, XGBoost
Genetic SNP analysis for obesity [39]	Uses ML to suggest personalized meal plans based on caloric needs, lifestyle habits	Genetic risk evaluation	SVM, decision trees
Smart wearable for continuous monitoring [68]	Tracks physical activity, sleep, and caloric intake; provides real-time feedback for lifestyle adjustments	Continuous lifestyle monitoring	CNN, recurrent neural networks
Preoperative obesity risk assessment [69]	Assesses risks like respiratory issues in obese patients undergoing surgery	Perioperative risk prediction	Extreme gradient boosting, ANN

**Table 3:** Utilization of different machine and deep learning models in bariatric surgery.

Authors	Objective	Machine Learning Models	Remark
Assaf et al. [86]	To predict preoperative hiatal hernia diagnosis.	DT. Dataset: Anthropometric and obesity-related comorbidities data collected (2012–2015) before bariatric surgery for HH. Sensitivity of method SS 38.5% and DT 60.2%	The decision tree model performed better in accuracy and sensitivity than the swallow study (barium swallow X-ray study).
Cheng et al. [87]	This study used phone sensors to predict pulmonary function, employing a universal support vector machine model that processes signal and patient demographic features to determine function categories.	SVM. Dataset: 35 patients NorthShore University, 6-min walk test (6WMT) data recorded for pulmonary function on smartphones. Global Initiative for Chronic Obstructive Lung Disease (GOLD) GOLD 1-IV classified with an accuracy of 98.51–100%.	SVM had an accuracy of 99%.
Gao et al. [88]	This research described a method for diagnosing obstructive sleep apnea syndrome utilizing ballistocardiogram data and ML.	SVM and fusion of LR-SVM Data: Ballistocardiogram (BCG) signals were captured via piezoelectric ceramic sensors placed under a mattress. LR-SVM was diagnosed with sensitivity, specificity, and accuracy values of 74%, 75%, and 75%, respectively.	LR-SVM performs relatively better than SVM alone.
Liew et al. [89]	This study analyzed the prevalence and risk factors of gallbladder disease in obese individuals using logistic regression and ANN.	LR and ANN. Data: 117 obese patients underwent bariatric surgery and cholecystectomy from 1999 to 2005 in Taiwan. ANN classified with an accuracy of 97.14% and type II error of 25% (gallstone cases as non-gallstone) and LR with 88.2% accuracy and 100% error.	ANN demonstrated a better classification rate compared to LR.
Mencar et al. [78]	To predict the severity of obstructive sleep apnea syndrome using ML.	MV, NB, KNN, CT, RF, SVM, AdaBoost-SVM, ML, LR, SVR, AdaBoost-SVR Data: 313 patients of OSAS. SVM: 65% AUC, 44.7% CA, 44.7 recall. RF: 63.7% AUC, 44.1% CA, 44.1% recall.	In classification and regression, SVM gave better accuracy and AUC, but RF performed better in precision or recall.
Razzaghi et al. [90]	This study examined the effectiveness of unbalanced classification algorithms in predicting outcomes for type 2 DM obese patients who have undergone bariatric surgery.	NB, RBFNN, KNN, DT, J48, SVM, and LR Data: Premier Healthcare Database. Predicting diabetes (using bagging with chi-squared): G-mean of 0.84 and ROC curve of 0.91. Predicting angina and heart failure (using RF with Chi-squared): G-mean of 1.0 and ROC 1.0.	
Twinanda AP [82]	This research introduced RSDNet, a deep-learning pipeline that automatically calculates the remaining operation duration (RSD) during surgery using visual input from laparoscopic films.	Naive and DL-RSDNet. Data: Cholec (120 laparoscopic cholecystectomy videos) and bypass (170 laparoscopic gastric bypass videos). Cholec120 dataset: Naïve (MEA 18.1 min and RSDNet (MEA 11 min). Bypass170 dataset: Naïve (MAE ~30.5 min) and RSDNet (MAE ~15.7 min).	In medium surgery, Naive and RSDnet perform comparably, but in short and long surgery, RSDnet performed much better.
Viswanath et al. [80]	To assess the accuracy of smartphone-based spirometry using ML.	NB, KNN, LR, RF, GB, CNN, RNN. Data: 36,161 audio recordings related to smartphone spirometry. GB: 97.8% precision and 91.6% recall. RNN: 98.3% precision and 88% recall.	GB and RNN performed with better precision and recall compared to others.
Zhou et al. [69]	This study evaluated six ML algorithms to predict difficult intubation in obese patients.	LR, TR, RF, GBm, GBdt, and XGBc. Data: 1015 obese patients from the public database. Xgb: Acc. > 80%, Prec. 100%, AUC 0.736. Gbdt: Prec. 71–100% and AUC 0.72–0.78.	Xgbc algorithm outperformed the others in accuracy (80%) and precision (100%).

**Table 4:** Recommendations based on key findings.

Limitation	Recommendation
AI algorithms rely on huge, high-quality datasets to generate accurate predictions. Inconsistent or incomplete data can result in incorrect outcomes.	Collecting complete and high-quality data, including diverse demographic information, can improve the accuracy and fairness of AI forecasts [102].
AI models, particularly complicated ones like neural networks, can overfit training data, rendering them less successful on fresh, unseen data.	Continuously assessing and updating AI models with new data might assist reduce overfitting and increase generalizability [103].
Many AI models, particularly deep learning models, are frequently regarded as “black boxes”, making it difficult to comprehend how they arrive at precise predictions.	Developing approaches for making AI models more interpretable can help healthcare providers comprehend and trust AI-driven suggestions [104].
The utilization of personal health data creates serious ethical and privacy concerns. Ensuring data security and patient consent is critical.	Integrate AI into healthcare by training people, developing interdisciplinary teams, and establishing ethical frameworks for transparency and data privacy [101].
